# Epidemiology of *Mycobacterium abscessus* in England: an observational study

**DOI:** 10.1016/S2666-5247(21)00128-2

**Published:** 2021-10

**Authors:** Samuel Lipworth, Natasha Hough, Natasha Weston, Berit Muller-Pebody, Nick Phin, Richard Myers, Stephen Chapman, William Flight, Eliza Alexander, E Grace Smith, Esther Robinson, Tim E A Peto, Derrick W Crook, A Sarah Walker, Susan Hopkins, David W Eyre, Timothy M Walker

**Affiliations:** a Nuffield Department of Medicine, University of Oxford, Oxford, UK; b Big Data Institute, Nuffield Department of Population Health, University of Oxford, Oxford, UK; c Oxford University Hospitals NHS Foundation Trust, Oxford, UK; d National Mycobacterial Reference Service-Central and North, Public Health England, Public Health Laboratory, Birmingham, UK; e Tuberculosis, Acute Respiratory, Gastrointestinal, Emerging and Zoonotic Infections and Travel Migrant Health Division, National Infection Service, Public Health England, London, UK; f National Mycobacterial Reference Service-South, Public Health England, London, UK; g NIHR Oxford Biomedical Research Centre, John Radcliffe Hospital, Oxford, UK; h Oxford University Clinical Research Unit, Ho Chi Minh City, Viet Nam

## Abstract

**Background:**

*Mycobacterium abscessus* has emerged as a significant clinical concern following reports that it is readily transmissible in health-care settings between patients with cystic fibrosis. We linked routinely collected whole-genome sequencing and health-care usage data with the aim of investigating the extent to which such transmission explains acquisition in patients with and without cystic fibrosis in England.

**Methods:**

In this retrospective observational study, we analysed consecutive *M abscessus* whole-genome sequencing data from England (beginning of February, 2015, to Nov 14, 2019) to identify genomically similar isolates. Linkage to a national health-care usage database was used to investigate possible contacts between patients. Multivariable regression analysis was done to investigate factors associated with acquisition of a genomically clustered strain (genomic distance <25 single nucleotide polymorphisms [SNPs]).

**Findings:**

2297 isolates from 906 patients underwent whole-genome sequencing as part of the routine Public Health England diagnostic service. Of 14 genomic clusters containing isolates from ten or more patients, all but one contained patients with cystic fibrosis and patients without cystic fibrosis. Patients with cystic fibrosis were equally likely to have clustered isolates (258 [60%] of 431 patients) as those without cystic fibrosis (322 [63%] of 513 patients; p=0·38). High-density phylogenetic clusters were randomly distributed over a wide geographical area. Most isolates with a closest genetic neighbour consistent with potential transmission had no identifiable relevant epidemiological contacts. Having a clustered isolate was independently associated with increasing age (adjusted odds ratio 1·14 per 10 years, 95% CI 1·04–1·26), but not time spent as an hospital inpatient or outpatient. We identified two sibling pairs with cystic fibrosis with genetically highly divergent isolates and one pair with closely related isolates, and 25 uninfected presumed household contacts with cystic fibrosis.

**Interpretation:**

Previously identified widely disseminated dominant clones of *M abscessus* are not restricted to patients with cystic fibrosis and occur in other chronic respiratory diseases. Although our analysis showed a small number of cases where person-to-person transmission could not be excluded, it did not support this being a major mechanism for *M abscessus* dissemination at a national level in England. Overall, these data should reassure patients and clinicians that the risk of acquisition from other patients in health-care settings is relatively low and motivate future research efforts to focus on identifying routes of acquisition outside of the cystic fibrosis health-care-associated niche.

**Funding:**

The National Institute for Health Research, Health Data Research UK, The Wellcome Trust, The Medical Research Council, and Public Health England.

## Introduction

*Mycobacterium abscessus* pulmonary disease can be devastating in patients with cystic fibrosis. This pathogen is highly antibiotic-resistant and treatment is challenging. *M abscessus* pulmonary disease (caused by one of three subspecies: *massiliense, abscessus*, and *bolletii*) can be progressive and incurable and is a relative contraindication to lung transplantation. In common with other non-tuberculous mycobacteria, acquisition of *M abscessus* was until recently considered only to occur from the environment, especially from contaminated water sources.^1^, ^2^

Several studies in cohorts of patients with cystic fibrosis have described genomically almost identical isolates from patients with potential opportunities for cross-infection.^3^, ^4^, ^5^ A large global study showed multiple internationally distributed dominant clades which accounted for most infections in patients with cystic fibrosis.^3^ The authors hypothesised that widespread recent transmission, most likely indirect person-to-person transmission through environmental contamination (eg, via fomites or aerosols) in health-care settings and other shared venues was the most likely explanation. Based on these studies, international guidelines suggest that person-to-person transmission might be an important mechanism for *M abscessus* acquisition in patients with cystic fibrosis.^6^, ^7^ However, smaller reports have not substantiated this hypothesis.^8^, ^9^, ^10^ No studies have investigated the genomic epidemiology of *M abscessus* from patients without cystic fibrosis.

**Research in context**

**Evidence before this study**

We searched PubMed for studies published from database inception to Dec 1, 2020, with no language restrictions, using the terms “Mycobacterium abscessus”, “transmission”, and “whole genome sequencing”. All studies published to date have focussed on the molecular epidemiology of *M abscessus* in patients with cystic fibrosis. The largest study to date identified multiple internationally distributed dominant clones that were responsible for most infections in patients with cystic fibrosis. Several studies have found evidence of highly genomically related strains among patients attending the same cystic fibrosis centre, raising concern about cross-infection. However, three smaller studies have identified genomic clusters of isolates from cystic fibrosis patients who have no epidemiological connections and concluded there was no evidence of cross-transmission. Although some uncertainty exists in the literature, the predominating interpretation of the available data is that there is a substantial risk of cross-transmission, something reflected in international guidelines.

**Added value of this study**

The dataset in this study (unlike most previous studies) is unselected and sequential, including isolates from all patients (irrespective of underlying diagnosis) over a 5-year period. By linking the largest genomic dataset assembled to date with a national health-care usage database, we have unprecedented ability to resolve potential transmission events. We show that *M abscessus* isolates from patients with cystic fibrosis are often highly genomically similar to those with other, or no, chronic respiratory disease. These clusters are widely geographically distributed and, in keeping with this observation, we show that most patients with similar isolates have no identifiable epidemiological links. We found a low risk of household transmission, further implying that the risk of transmission associated with short-term nosocomial exposure is likely to be low, a finding supported by our regression analysis.

**Implications of all the available evidence**

Earlier studies, which only analysed isolates from patients with cystic fibrosis, suggested genomic clusters were propagated by (probably indirect) transmission among patients with cystic fibrosis in health-care facilities. Our study challenges this interpretation by showing that genomic clusters of M abscessus are widely geographically dispersed and shared across all patient groups. Short-term nosocomial exposure with normal infection control procedures is likely to carry a low risk of person-to-person transmission. Future efforts to protect patients from infection should focus on identifying potential locally or nationally distributed vectors.

A clear understanding of cross-infection risk is crucial to protect patients with cystic fibrosis in health-care facilities. Although nosocomial transmission needs to be minimised, interventions might also cause financial and operational challenges and psychological harm for patients. Over the past 6 years, Public Health England (PHE) has implemented whole-genome sequencing (WGS) to replace existing reference laboratory techniques for all mycobacteria, producing a near-complete dataset of all sequenced *M abscessus* clinical isolates in England from patients with and without cystic fibrosis. These data have been linked to routinely collected health-care usage datasets, presenting an opportunity to investigate person-to-person transmission of *M abscessus* on a national scale across all patient groups.

## Methods

### Study design and sample collection

In this observational study, we did a retrospective analysis of routinely collected *M abscessus* WGS data in England. All mycobacterial isolates are sent to one of two PHE reference laboratories in London and Birmingham for WGS. Routine sequencing began in Birmingham for the Midlands at the beginning of February, 2015, and for the north of England October, 2016; it began in London for the south of England in January, 2018. All isolates from these time points until Nov 14, 2019, are included, in addition to all available isolates sequenced before the routine service began (n=10). Historical records of unsequenced isolates (patient details and collection date) were available from November, 1997, from Birmingham and from November, 2001, from London.

This study was done as a public health investigation with internal approval from PHE and therefore ethics committee approval was not required.

### Sequencing and bioinformatics

WGS was done by PHE as part of the routine clinical service on an Illumina Miseq instrument (San Diego, CA, USA) as previously described.^11^ Reads were mapped to a reference genome (National Center for Biotechnology Information reference sequence NC_010397·1) using the PHE bioinformatics pipeline v1.0.2 and sequences were compared using recombination-adjusted (using ClonalFrameML v1.11),^12^ maximum likelihood phylogenies (IQTree v1.6.12);^13^ single nucleotide polymorphism (SNP) distances; and time-scaled phylogenies and molecular clock estimation (BEAST v1.10.4; appendix p 2).^14^ Within-patient diversity was estimated using all sequenced isolates for patients for whom more than one of these were available. We also did a sensitivity analysis to determine the effect of choice of reference (appendix p 3). Clusters of isolates potentially consistent with recent transmission were identified using the previously defined genomic threshold of fewer than 25 SNPs.^4^

### Epidemiological linkage

Laboratory records were linked to the national Hospital Episode Statistics (HES) database using patient-specific identifiers; data were extracted detailing health-care contact, clinical procedures, and diagnostic codes (appendix p 3). Underlying diagnoses were identified using codes submitted before the date of first isolation of *M abscessus*. Respiratory diagnoses were assigned hierarchically (appendix p 7). For epidemiological analysis, we assumed that isolates submitted to the reference laboratories before WGS was introduced would belong to the same clusters as subsequently sequenced isolates from the same patients.

We examined whether epidemiological contact with another patient (defined as attendance to the same unit on the same day or shared postcode district [approximately 2066 in England] or primary health-care practice in the year before acquisition) was associated with the acquisition of genetically similar isolates. We adopted the model of Bryant and colleagues,^4^ assuming that patients could become infected with *M abscessus* up to 1 year before first isolation and remained potentially infectious from this point onwards. We used the date of first isolation of *M abscessus* (whether sequenced or not) for the epidemiological analysis and considered the first isolate per cluster per patient (ie, if a patient had isolates in multiple clusters the first from each was included).

To identify possible household contacts we searched the health-care usage database (HES) to identify patients with cystic fibrosis who lived at the same postcode as a cystic fibrosis patient in our dataset in the year in which *M abscessus* was first isolated from them. Although the mean number of households within a postcode is approximately 15, the population prevalence of cystic fibrosis is such that most pairs of individuals sharing the same full postcode and both with cystic fibrosis would be expected to be in the same household. Where these pairs were also both present in our laboratory records (hence had both had *M abscessus* isolated) and shared a surname, we defined these as siblings. One possible sibling pair was identified in which individuals shared a surname but were not household contacts in the year of acquisition (but had been previously).

### Geospatial analysis

For each patient, the postcode (typically shared by approximately 15 properties) closest in time to their first *M abscessus* isolate (whether sequenced or not) was used. We identified high-density phylogenetic clusters using TreeGubbins and for each calculated the ratio of the median genetic distance within and between geographical areas (Nomenclature of Territorial Units for Statistics [NUTS] regions identified using patient postcodes; appendix p 3). We did a permutation test^15^ to determine whether observed values were compatible with the null hypothesis of no regional clustering of isolates (appendix p 3). We additionally calculated the Pearson correlation between distance to nearest genomic neighbour (in SNPs) and geographical distance (measured using the Harversine distance) between patient's postcodes. We generated a phylogenetic tree including all isolates from this and three previous studies.^3^, ^9^, ^16^

### Statistical analysis

Descriptive statistical analysis of laboratory and HES data were done using R v3.4.3, as were all other analyses unless otherwise stated. Proportions of clustered isolates between subgroups (patients with *vs* without cystic fibrosis overall, patients with cystic fibrosis *vs* with bronchiectasis from 2014, patients with cystic fibrosis and bronchiectasis *vs* all other patients, patients with cystic fibrosis with a fist isolate after 2014 or 2015 *vs* before these years) were compared using Fisher's exact test. To determine whether the observed proportion of clusters containing only patients with cystic fibrosis was greater than that expected by chance alone, a permutation test was done by randomising the diagnostic labels 1000 times and recalculating the number of cystic fibrosis-only clusters (appendix p 2). For patients with cystic fibrosis, we additionally analysed whether the median genomic distance to the nearest patient with cystic fibrosis was smaller than the median genomic distance to the nearest patient without cystic fibrosis using a Wilcoxon rank-sum test. Univariate and multivariable regression analysis were done to investigate factors (health-care exposures, demographic or socioeconomic and clinical variables) associated with acquisition of a genomically clustered strain (ie, genomic distance from another genome <25 SNPs).^4^ For each factor included in the models, the odds ratio (OR; or adjusted OR [aOR] where relevant) and 95% CIs were calculated. Only the first isolate per patient was used. As it was unclear what relevant exposures might be, we considered this analysis to be exploratory and initially included all demographic, clinical, and socioeconomic factors available. To ensure that only variables making a significant contribution were included, the final model was fitted by backwards elimination using the Akaike Information Criteria, allowing for potential interactions and non-linearity (appendix p 4). We additionally did a secondary regression analysis using the same methodology to investigate factors associated with acquiring a clustered isolate in patients with cystic fibrosis. For all statistical tests we considered p<0·05 as the threshold for significance.

### Role of the funding source

The funder of the study had no role in study design, data collection, data analysis, data interpretation, or writing of the report.

## Results

2431 isolates were sequenced by the reference laboratory; full linkage was achieved for 2297 isolates from 906 patients (appendix p 8). The most common sample type was sputum (1997 [87%] of 2297) followed by bronchoalveolar lavage (135 [6%] of 2297). The most common non-respiratory sample types were blood (n=19 [<1%]), tissue of unknown source (n=14 [<1%]), and cutaneous biopsies (n=10 [<1%]; appendix p 17). The most common primary respiratory diagnosis was cystic fibrosis (408 [45%] of 906). Another 296 (33%) patients had a different chronic respiratory diagnosis and 202 (22%) had no documented respiratory diagnosis. These diagnostic groups were reflected in a bimodal age distribution (median age of patients with cystic fibrosis 21 [IQR 16–27] years *vs* patients without cystic fibrosis 65 [49–74] years; appendix p 18). Patients with cystic fibrosis had a higher number of isolates sequenced (median 2 [IQR 1–4] *vs* 1 [1–3]; p<0·0001).

We adopted the previously reported threshold of fewer than 25 SNPs for inferring possible recent transmission.^4^ We estimated a molecular clock of 1·1 SNPs per genome per year (95% highest posterior density interval 0·9–1·4), consistent with the mean time to most recent common ancestor for two clustered strains being approximately 10·9 years. We found that this threshold represented more than 95% of within-patient diversity (median time between first and last sequenced isolates 246 days [IQR 77–570]) in the same subspecies, which is consistent with previous studies (appendix p 9).^3^, ^4^

**Figure 1:**
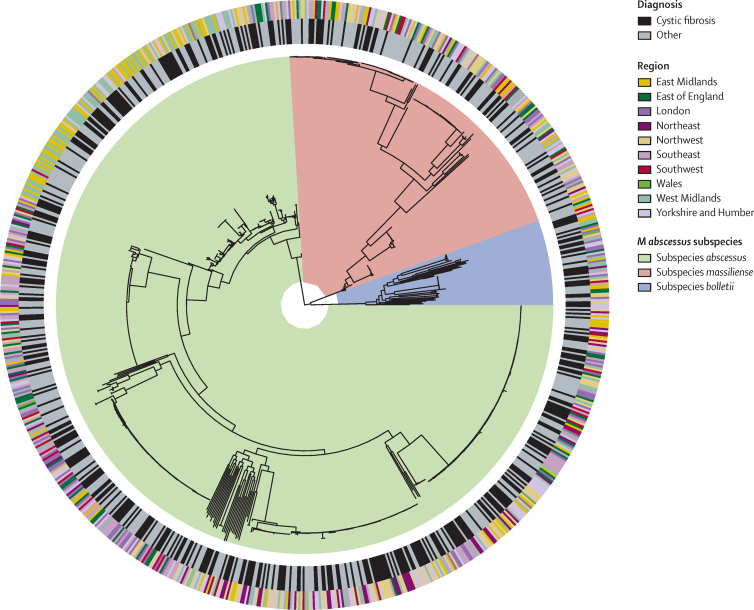
Phylogeny of 944 *Mycobacterium abscessus* isolates The phylogenetic tree includes one unique isolate per patient per cluster (36 were excluded due to missing data). The inner ring shows the presence or absence of a diagnosis of cystic fibrosis. The outer ring shows the geographical region of England in which the patient lives.

Retaining the first genome per patient per cluster, there were 703 *M abscessus s*ubspecies *abscessus*, 52 *M abscessus* subspecies *bolletii*, and 189 *M abscessus* subspecies *massiliense* isolates (figure 1). 560 (62%) of 906 patients had one or more isolate that was part of a genomic cluster (n=115 clusters, median size 2 [range 2–54]); 364 (40%) patients had non-clustered isolates. 32 (4%) patients had multiple isolates that fell into more than one cluster or had at least one clustered and one non-clustered isolate.

**Figure 2:**
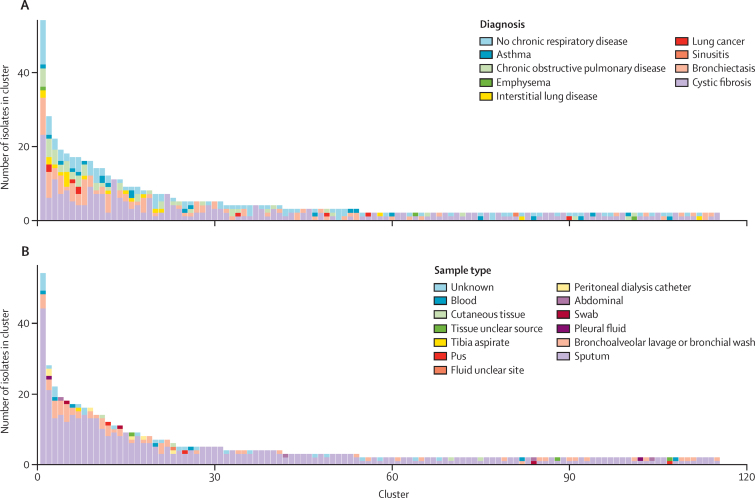
Distribution of *Mycobacterium abscessus* isolate cluster sizes by sample type and patient diagnosis Distribution of cluster sizes for all clusters identified using a genomic distance threshold of fewer than 25 SNPs, by diagnosis (A) and by sample types (B). The algorithm used to assign respiratory diagnoses to patients is in the appendix (p 7).

Patients with cystic fibrosis were no more likely than patients without cystic fibrosis to have a clustered isolate (258 [60%] of 431 *vs* 322 [63%] of 513; p=0·38). Comparing isolates from patients with cystic fibrosis whose first isolate occurred from 2014 (who likely underwent enhanced infection control and cohorting) with those from patients without cystic fibrosis but with bronchiectasis from the same period revealed no difference in the proportion which were clustered (227 [64%] of 354 *vs* 103 [69%] of 150; p=0·36). In some centres, cystic fibrosis and bronchiectasis services are co-located and both groups might have been subject to enhanced infection control procedures. We therefore repeated this analysis to compare patients with cystic fibrosis and bronchiectasis with all other patients; again, there was no difference (330 [65%] of 504 *vs* 204 [62%] of 327; p=0·37). Furthermore, when we looked only at patients with cystic fibrosis, the proportion with a clustered isolate was higher in those who acquired their first isolate after 2014 versus those who acquired it before then (227 [64%] of 354 *vs* 31 [40%] of 77; exact p<0·0002) and in those who acquired their first isolate after 2015 versus those who acquired it before then (218 [69%] of 318 *vs* 40 [43%] of 93; p<0·0003). High proportions of clustered patients were observed in non-sputum-producing respiratory phenotypes (eg, 20 [59%] of 34 patients with asthma, 9 [90%] of 10 patients with lung cancer, and 17 [81%] of patients with interstitial lung disease. Notably, four samples from cutaneous biopsies clustered with other patients with a variety of diagnostic codes (including cystic fibrosis or non-cystic fibrosis bronchiectasis; figure 2; appendix p 10).

92 (80%) of 115 clusters contained at least one patient with cystic fibrosis, 68 of which crossed disease strata (ie, contained at least one cystic fibrosis patient and one patient with a different or no respiratory diagnosis). We reasoned that if cystic fibrosis communities or health-care facilities are the primary facilitators of clonal outbreaks, then the number of clusters containing exclusively cystic fibrosis patients ought to be greater than would be expected by chance. This was not the case (observed proportion of exclusively cystic fibrosis clusters 0·21, permuted 95% CI 0·00–0·22). For patients with cystic fibrosis, the median genomic distance to the nearest patient with cystic fibrosis was 24 SNPs (IQR 9–51) whereas the median genomic distance to the nearest patient without cystic fibrosis was 31 SNPs (8–67, p=0·093).

**Figure 3:**
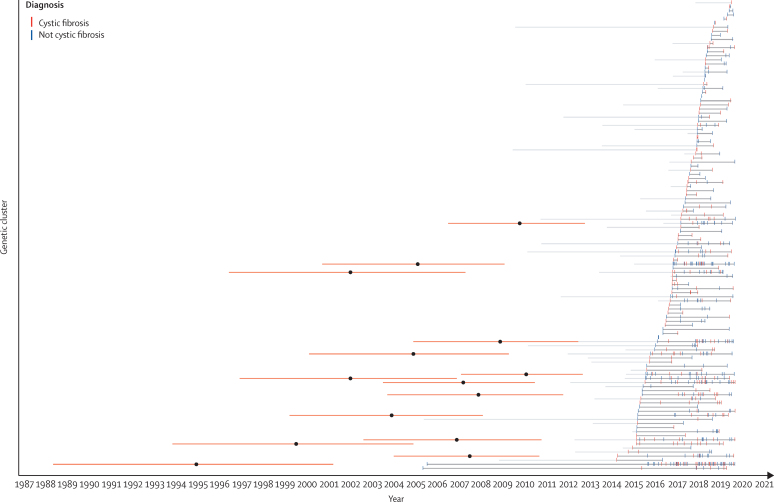
Timeline of genomic clusters identified in this study Isolates (deduplicated per cluster) are shown for patients with and without cystic fibrosis. Clusters of isolates defined using a genomic distance threshold of fewer than 25 SNPs are connected by dark grey lines. Ligh grey lines show the time to the earliest non-sequenced isolate belonging to a member of a given cluster. The orange bars show the 95% highest posterior density interval for the inferred date of the root for the time-scaled phylogenetic trees for larger clusters (n≥10); the mean point estimate of these dates is shown as a black dot.

Because there was a substantial change in infection control guidelines in late 2013,^17^ we considered the possibility that previously described clusters might have gradually died out after this period and therefore be unrelated to those described here. Bayesian dating analysis showed that all larger clusters probably emerged before this period (figure 3). We identified 107 patients in these clusters with previous (unsequenced) isolates pre-dating the implementation of enhanced infection control procedures and for the purposes of our epidemiological analysis assumed these would mostly fall in the same cluster as later sequenced isolates. As we analysed epidemiological data from the date of first acquisition (not sequencing date), if substantial person-to-person transmission was occurring before the introduction of the new guidelines we would have expected to have observed it.

For each measure of nosocomial contact (eg, outpatient attendances, days in hospital, respiratory procedures) we considered the total number of relevant exposures in the year preceding the first recorded isolate of *M abscessus* (whether sequenced or not) for each patient (table). Multivariable models revealed evidence of increasing age (aOR per 10 years 1·14, 95% CI 1·04–1·26) being associated with an increased risk of being colonised with a clustered isolate. There was some evidence of an association with increasing morbidity (aOR 1·02, 95% CI 1·00–1·04). When restricting only to patients with cystic fibrosis, there was some evidence of decreased risk of having a clustered isolate with increasing number of inpatient days (aOR per 7 days 0·94, 95% CI 0·88–1·00; p=0·045) and increasing risk associated with greater comorbidity (adjusted OR 1·03, 95% CI 1·00–1·06; appendix 21). Notably, in both analyses neither the number of outpatient attendances nor inpatient days were significantly associated with the risk of having a clustered isolate in univariate analysis.

**Table:** Multivariable predictors of acquiring a clustered *Mycobacterium abscessus* isolate

		**Patients with non-clustered isolates (n=342)**	**Patients with clustered isolates (n=519)**	**OR (95% CI, p value)**	**aOR (95% CI, p value)**
Sex					
	Female (n=427)	161 (37·7%)	266 (62·3%)	..	..
	Male (n=434)	181 (41·7%)	253 (58·3%)	0·85 (0·64–1·11, p=0·23)	..
Age, years		30 (20–63)	39 (22–69)	1·10 (1·04–1·16, p=0·0014) per 10 years	1·14 (1·04–1·26, p=0·0071)per 10 years
Outpatient attendances		9 (4–16)	10 (5–15)	1·01 (0·99–1·02, p=0·39)	..
Inpatient days		1 (0–14)	2 (0–14)	0·99 (0·95–1·04, p=0·68) per 7 days	..
Elixhauser score		3 (0–10)	5 (2–13)	1·03 (1·02–1·05, p<0·0003)	1·02 (1·00–1·04, p=0·058)
Respiratory procedures		0 (0–0)	0 (0–1)	1·20 (1·04–1·41, p=0·019)	1·15 (0·98–1·36, p=0·093)
Rural or urban dwelling					
	Hamlet (n=22)	10 (45·5%)	12 (54·5%)	..	..
	Town and fringe (n=73)	30 (41·1%)	43 (58·9%)	1·19 (0·45–3·13, p=0·72)	..
	Urban (n=703)	277 (39·4%)	426 (60·6%)	1·28 (0·53–3·01, p=0·57)	..
	Village (n=63)	25 (39·7%)	38 (60·3%)	1·27 (0·47–3·38, p=0·64)	..
Index of multiple deprivation decile					
	Most deprived 10% (n=99)	44 (44·4%)	55 (55·6%)	..	..
	More deprived 10–20% (n=102)	44 (43·1%)	58 (56·9%)	1·05 (0·60–1·84, p=0·85)	..
	More deprived 20–30% (n=81)	36 (44·4%)	45 (55·6%)	1·00 (0·55–1·81, p=1·00)	..
	More deprived 30–40% (n=67)	21 (31·3%)	46 (68·7%)	1·75 (0·92–3·40, p=0·091)	..
	More deprived 40–50% (n=95)	37 (38·9%)	58 (61·1%)	1·25 (0·71–2·23, p=0·44)	..
	Less deprived 50–60% (n=70)	26 (37·1%)	44 (62·9%)	1·35 (0·73–2·55, p=0·34)	..
	Less deprived 60–70% (n=73)	25 (34·2%)	48 (65·8%)	1·54 (0·83–2·89, p=0·18)	..
	Less deprived 70–80% (n=87)	30 (34·5%)	57 (65·5%)	1·52 (0·84–2·77, p=0·17)	..
	Less deprived 80–90% (n=103)	46 (44·7%)	57 (55·3%)	0·99 (0·57–1·73, p=0·98)	..
	Least deprived 10% (n=84)	33 (39·3%)	51 (60·7%)	1·24 (0·69–2·24, p=0·48)	..
Diagnosis					
	Bronchiectasis (n=146)	48 (32·9%)	98 (67·1%)	..	..
	No chronic respiratory diagnosis (n=180)	89 (49·4%)	91 (50·6%)	0·50 (0·32–0·78, p<0·0027)	0·66 (0·41–1·07, p=0·10)
	Asthma (n=34)	14 (41·2%)	20 (58·8%)	0·70 (0·33–1·53, p=0·36)	0·83 (0·38–1·84, p=0·64)
	Lung cancer (n=10)	1 (10·0%)	9 (90·0%)	4·41 (0·79–82·46, p=0·17)	2·89 (0·50–54·7, p=0·33)
	Cystic fibrosis (n=400)	165 (41·2%)	235 (58·8%)	0·70 (0·47–1·03, p=0·077)	1·27 (0·72–2·26, p=0·41)
	Chronic obstructive pulmonary disease (n=70)	21 (30·0%)	49 (70·0%)	1·14 (0·62–2·14, p=0·67)	1·03 (0·56–1·94, p=0·93)
	Interstitial lung disease (n=21)	4 (19·0%)	17 (81·0%)	2·08 (0·72–7·53, p=0·21)	1·88 (0·64–6·89, p=0·29)

Data are n (%), median (IQR), or OR (95% CI, p value). 45 patients had one or more incomplete datapoint and were excluded from the model. Univariate estimates (ORs) are shown for all variables, multivariable estimates (aOR) are only shown for variables included in the final model. Inpatient days, outpatient attendances, and respiratory procedures refer to the number of these in the year before *M abscessus* was first isolated from the patient. OR=odds ratio. aOR=adjusted OR.

If contact with a contaminated environment is a strong risk factor for *M abscessus* transmission, then household contacts who both have cystic fibrosis and *M abscessus* infection would be expected to be colonised with the same strain. In our dataset there were three such pairs of siblings: two had genetically divergent strains (14 103 and 17 352 SNPs difference), whereas one had near identical strains (2 SNPs). There was a further possible sibling pair with divergent strains (54 878 SNPs; appendix p 4), although this pair were not household contacts at the point of acquisition. There were additionally 25 pairs of possible household contacts who both had cystic fibrosis but one had not had *M abscessus* infection. Given that we obtained the full records of both English reference laboratories and that none of these patients have a recorded diagnosis of mycobacterial infection, it is unlikely that they had experienced disease due to *M abscessus*.

Only 47 (13%) of 356 isolates with a nearest genetic neighbour potentially compatible with transmission (<25 SNPs) had a plausible epidemiological contact. In these cases, person-to-person transmission (direct or indirect) cannot be excluded (appendix pp 11–12). Of these, 33 were cystic fibrosis patients meaning that 33 [8%] of 408 patients with cystic fibrosis in this study had an isolate that could have been acquired by person-to-person transmission.

**Figure 4:**
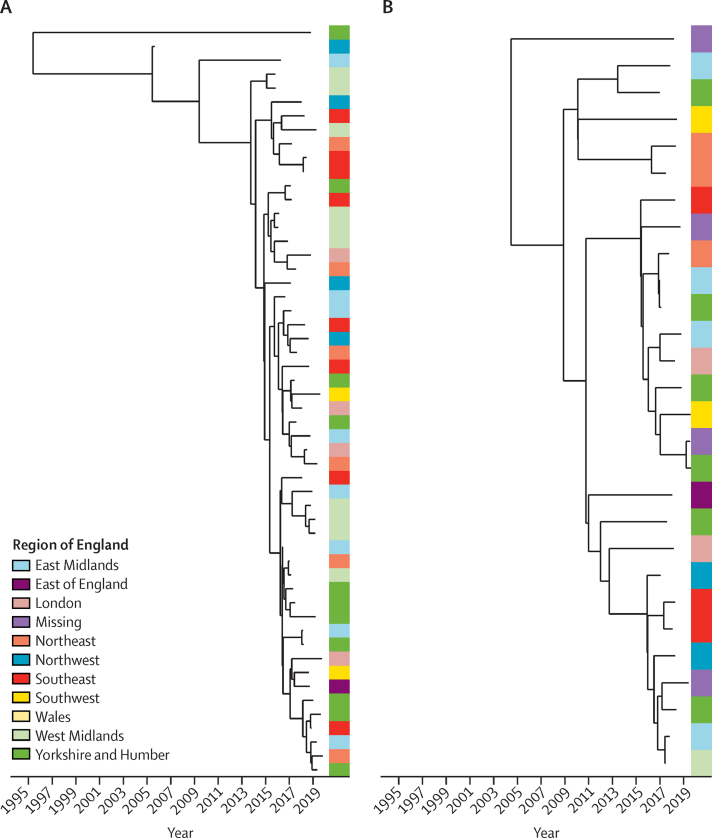
Dated phylogenies and geographical distribution of *Mycobacterium abscessus* isolates in the two largest clusters in this study Clusters were identified using a genomic distance threshold of fewer than 25 SNPs. The side panel shows the region of England in which the patient lived at the time of isolate collection. Dated phylogenies for all clusters are shown in the appendix (p 13).

872 (96%) of 906 patients had available postcodes and could be linked to one of nine geostatistical regions in England (appendix p 3). Larger clusters (isolates from ten or more patients) linked by a genomic distance of fewer than 25 SNPs were not confined to particular geographical regions (median 7 regions, range 5–9; figure 4, appendix p 13). All but one larger cluster contained patients both with and without cystic fibrosis. As SNP clusters are based on an arbitrary threshold, we did an additional whole-phylogeny-based test to confirm this observation. We compared ratios of genomic distances within and between geostatistical regions for each high-density phylogenetic cluster (appendix p 22). In all cases the observed within-to-between region ratio was compatible with chance. We observed no correlation between distance to nearest genomic neighbour and geographical proximity (Pearson coefficient 0·03) and found that distance to nearest genomic neighbour was smaller between isolates in different versus the same geographical (NUTS) regions (median 15 [IQR 4–46] *vs* 3272 SNPs [115–16 618]; p<0·0001). Furthermore, apart from samples from one study,^16^ we observed that the genomes of isolates obtained from previous global studies were distributed throughout the phylogeny of English isolates obtained in this study (appendix p 14).

## Discussion

We analysed consecutive, unselected genomic data linked to health-care records to investigate the extent of person-to-person transmission of *M abscessus* in England. Genomic clusters of *M abscessus* are not disease-specific; only a minority are exclusive to cystic fibrosis. As has previously been shown to be the case for patients with cystic fibrosis, most patients without cystic fibrosis in England are colonised with a clustered isolate. This situation is contrary to what would be expected if, as currently postulated, outbreak strains are primarily propagated in nosocomial cystic fibrosis environments. It was particularly notable that several patients with non-respiratory isolates (eg, skin abscesses, bone aspirates, or peritoneal dialysis fluid) had genomically near-identical isolates to respiratory patients. We observed no geographical structure to the phylogeny and in keeping with this, by linking genomic data to a detailed national epidemiological database, showed that most patients in clusters have no identifiable epidemiological links. Previous studies sequenced selected stored isolates exclusively from groups of patients with cystic fibrosis and therefore do not represent the full landscape of *M abscessus* epidemiology. Our findings have important implications for future efforts to protect patients.

Current international guidelines state that “person-to-person transmission may be an important mechanism in the acquisition of *M abscessus*, at least in cystic fibrosis patients”.^6^, ^7^ A common source of clinical concern occurs where *M abscessus* is isolated from several patients attending the same clinic on the same day. In most of such cases in this study, the genomes sequenced were unrelated, effectively excluding person-to-person transmission. Although we identified a small number of cases (approximately 5% of patients) in which the genomic relatedness of isolates and possible epidemiological connections could be compatible with person-to-person transmission, this number represents an upper bound estimate of the true extent of transmission given the integrated service design of regional cystic fibrosis networks and the high number of epidemiological contacts therefore expected by chance. It is hard to explain how patients who live in geographically separate regions, do not access the same health-care facilities, and are unlikely to share social connections could transmit, even indirectly, between each other. Unless there is a significant reservoir of healthy and asymptomatic carriers in the general population, it is unlikely that widespread person-to-person transmission explains our observations. We hypothesise that national dissemination via a widely distributed, possibly water-associated exposure, could be compatible with our data given what is known about the environmental ecology of non-tuberculous mycobacteria and the wide geographical distribution of clusters we observe. A prominent example of this has been shown for *M chimaera*,^18^ although our data would suggest that the relevant exposure for *M abscessus* is more commonly encountered in the community.

Our regression analysis identified age as being significantly associated with the risk of acquiring a clustered strain but there was no association with increased hospital contact. Older and multi-comorbid patients might be repeatedly exposed to clustered strains via some unknown environmental vector whereas non-clustered strains are sporadically found in a highly diverse environmental pool that these patients are less exposed to. The finding that patients with cystic fibrosis appear to be marginally protected from acquisition of a clustered isolate with increasing time spent as an inpatient is likely to be spurious but might be explained by reduced exposure if these strains are primarily community acquired. Although we identified one pair of siblings in whom household transmission might have occurred, the rarity of transmission amongst individuals with probable household exposure suggests that transmission from short-term nosocomial exposure would be even rarer, which is consistent with our observations at the national level. Nevertheless some highly infectious patients could possibly transmit to other patients, especially in the case of a breakdown in infection control procedures.

PHE's systematic sequencing of non-tuberculous mycobacteria began in 2015 and after high-profile reports in the literature of *M abscessus* outbreaks and subsequent enhanced infection control procedures.^4^ Our findings could therefore be interpreted as representing evidence of the effectiveness of these measures; however, we think this explanation is unlikely. Stringent infection control measures and the principle of segregating patients with cystic fibrosis from each other were in place long before the introduction of these enhanced procedures.^19^ Most new clusters are still caused by clustered isolates. Bayesian dating analysis of larger clusters revealed that these arose before the introduction of enhanced infection control procedures, suggesting that these had minimal efficacy to disrupt their prorogation. Furthermore, if transmission was predominantly associated with health-care settings before the introduction of these measures and subsequent interventions effective, we would expect the incidence of cases in patients with cystic fibrosis to change significantly; this has not been observed.^17^ We might also expect a divergence in the epidemiology of *M abscessus* between patients with and without cystic fibrosis (particularly those with non-cystic fibrosis bronchiectasis). No such difference was observed.

Studies that only sequence isolates during suspected outbreaks or isolates selected for storage risk bias. The relatively unselected nature of our patient population is a key strength of this study. Limitations include possible ascertainment bias leading to over-representation of patients with cystic fibrosis due to heightened awareness of *M abscessus* infection in this community in recent years, the non-availability of sequences for pre-2015 isolates, and our assumption that patients remain colonised with the same strain. The use of an arbitrary SNP threshold is an additional limitation but also permits direct comparison with previous studies and reflects current public health practice.

In summary, the observation in this and other studies of widely disseminated genetically near-identical clones is striking, but crucially these are not restricted to patients with cystic fibrosis. It is difficult to explain how cross-transmission could have led to the widespread geographical dispersion of clonal lineages we have observed among patients, the vast majority of whom have no epidemiological links. Although it is possible that these clones are asymptomatically carried by a much wider population than previously thought, it seems more probable that an as yet unidentified, widely distributed, environmental vector might underlie *M abscessus* clusters in patients with chronic respiratory disease (not just cystic fibrosis). Our data clearly show that future studies and infection control approaches must consider a wider focus than exclusively a cystic fibrosis health-care-associated niche. The identification in this study and others of possible cross-transmission events warrants ongoing genomic surveillance and is one of many factors justifying high levels of infection control within facilities that treat patients with cystic fibrosis. These data should, however, also provide reassurance to clinicians and patients and their families that the risk of acquisition of *M abscessus* from other patients in health-care settings is low. These data also underline the value of unselected sampling frames when making inferences on the basis of molecular epidemiology.

## Data sharing

All sequencing data is available under NCBI project accession number PRJEB43019. The phylogenetic tree of 2297 isolates used in the analysis is available online.

## Declaration of interests

DWE declares grants from Robertson Foundation and lecture fees from Gilead, outside the submitted work. TMW is a Wellcome Trust Clinical Career Development Fellow (214560/Z/18/Z). All other authors declare no competing interests.
