# Supplementary material for: Epidemiology of Mycobacterium abscessus in England: an observational study
Source: Lancet Microbe. 2021 Oct;2(10):e498–507. doi: 10.1016/S2666-5247(21)00128-2 (PMC8481905; doi:10.1016/S2666-5247(21)00128-2)
Supplement: Supplementary appendix [file mmc1.pdf]

THE LANCET
Microbe

Supplementary appendix

This appendix formed part of the original submission and has been peer reviewed. We post it as supplied by the authors.

Supplement to: Lipworth S, Hough N, Weston N, et al. Epidemiology of *Mycobacterium abscessus* in England: an observational study. *Lancet Microbe* 2021; published online July 14. https://doi.org/10.1016/S2666-5247(21)00128-2.

Supplementary Appendix

Contents

Supplementary Methods ... 2
Sequencing ... 2
Recombination correction sensitivity analysis ... 2
Permutation test for clusters exclusive to CF patients ... 2
Choice of reference ... 3
Global Phylogeny ... 3
Geospatial analysis ... 3
Statistics ... 4
Patients in the same postcode ... 4
Supplementary Results ... 4
Additional potential sibling pair ... 4
References ... 4
Figure S1 ... 7
Figure S2 ... 8
Figure S3 ... 9
Figure S4 ... 10
Figure S5 ... 11
Figure S6 ... 12
Figure S8 ... 14
Figure S9 ... 15
Figure S10 ... 16
Supplementary Tables ... 17
Table S1 ... 17
Table S2 ... 19
Table S3 ... 20
Table S4 ... 22
Table S5 ... 22

Supplementary Methods

Sequencing

Clinical samples were cultured in BD Bactec MGIT liquid mycobacterial growth indicator tubes, from which an aliquot was removed to be prepared for sequencing. Sequencing was performed on Illumina instruments (MiSeq in Birmingham, HiSeq/NextSeq in London) as previously described[1]. The median sequencing depth was 63.6 (IQR 46.9-79.0). Median reference coverage was 91.6% (IQR 90.0-92.4). Stampy v1.0.17[2] was used to map reads to a the reference genome (NC 010397.1). Samtools Mpileup v0.1.181[3] was used to make variant calls with a minimum read depth of 5x and at least one read on each strand. Phylogenetic trees were computed using a generalised time reversible substitution model implemented with IQTree[4]. The input to IQtree was a core SNP alignment (created using SNP-sites[5]) of 562,704 positions which was padded with reference bases at invariant sites to create an alignment of total length 5067172; this was used as input to IQ. Branch lengths were corrected for recombination using ClonalFrameML v1.12 with default parameters[6]. The tree of 2297 isolates used in the analysis has been deposited at https://doi.org/10.6084/m9.figshare.14153219.v1. To test the strength of temporal signal, a permutation test (based on random permutations of sampling dates) was performed using the R package BactDating[7] (100000 permutations, $p=0.036$). A Time-scaled phylogeny were created with BEAST v1.10.13[8] using core genome alignments with recombination removed. Three independent runs were performed with a chain length of 100,000,000, of which the first 10% were discarded as burn-in. After ensuring similar convergence of runs, the log files were combined using LogCombiner/Tracer. The combined effective sample size was > 1000. From this output we also estimated the molecular clock which we reported as mean mutations per site per year with a corresponding 95% highest posterior density (HPD) interval. Clustering on a 25 SNP threshold was used to identify groups of isolates potentially consistent with recent transmission/point source acquisition. TreeGubbins[9] was used to identify high density phylogenetic clusters. As previously described, TreeGubbins identifies areas of high density in the phylogeny by comparing the observed density of each node (mean descendent branch length) to the expected density (mean branch length of remaining tree)[10].

Recombination correction sensitivity analysis

We compared the distributions of recombination corrected and non-corrected SNP distances for isolates with a nearest genomic neighbour <100 SNPs (comparing only the nearest neighbour for each isolate to avoid double counting) which revealed very similar distributions (recombination correct median 1 SNP, IQR 0-5 vs uncorrected median 1 SNP, IQR 0-7) and an overall Pearson correlation 0.77 $p<0.001$).

Permutation test for clusters exclusive to CF patients

We performed a permutation test to determine whether the observed proportion of clusters containing only CF patients was greater than that which would be expected by chance. Diagnostic labels were permuted and the proportion of clusters containing only CF

patients re-calculated. This procedure was repeated 1000 times. The test was determined to be compatible with the null hypothesis (that the ratio observed was compatible with chance), if the observed ratio fell within the 2.5-97.5 percentiles of the permuted null distribution.

Choice of reference

The reference we used (NC 010397.1) is M. abscessus subspecies abscessus, and so we considered the possibility that mapping to this may under-represent variation seen in M. abscessus subspecies massiliense clusters. To ensure that our analysis was robust to the fact that our pipeline used only a single M. abscessus subsp. abscessus reference, we re-analysed the original study of Bryant et al by mapping these raw reads to our reference; this did not affect the interpretation (Figures S9 and S10). We further mapped reads from all large (n>=10 patients) subspecies *massiliense* clusters in this study to a subspecies massiliense reference (NC018150.2). Clusters obtained from this analysis were identical to those obtained when mapping to the subspecies *abscessus* reference (data not shown).

Global Phylogeny

We included the genomes from all cluster-wise deduplicated isolates in this study as well as those from three prior global studies[10–12]. To avoid potential duplication we excluded isolates from the Bryant study from UK sites. A recombination corrected maximum likelihood phylogeny was constructed as above.

Geospatial analysis

For each patient in a cluster we identified the postcode from the Hospital Episode Statistics database (a database capturing many demographic and healthcare delivery related items for all hospitals in England) closest in time to that of their first isolate. These were converted to geographical coordinates using the ggMap package (v3.0.0) in R as an interface to the Google Maps API6. Postcodes were assigned to a Nomenclature of Territorial Units for Statistics (NUTS) using the postcodes.io api interfaced from R[13]. The nine NUTS 1 regions in England are: UKC – North East, UKD – North West, UKE – Yorkshire and the Humber, UKF – East Midlands, UKG – West Midlands, UKH – London, UKJ – South East, UKK – South West. High density phylogenetic clusters were identified using TreeGubbins[9]. For all clusters we quantified the median SNP distance between all isolates within and between NUTS regions and then generated a null distribution by random switching of NUTS regions. We performed 1000 permutations of this procedure to calculate the expected distribution of SNP ratios under the null hypothesis. We determined that there was significant within NUTS region clustering if the observed value was less than the 2.5 percentile in the null distribution.

Data Extracted from the Healthcare Episode Statistics Database (HES)

Linkage to HES was performed using NHS numbers from laboratory records. The following fields were extracted for all inpatient/outpatient episodes (where applicable): Treatment Specialty, Appointment date, Diagnosis, Procedure, Admission date, Discharge date, Treatment Site, Main specialty, GP practice, Postcode, Date of Birth, Rural/urban indicator, Sex and Index of Multiple Deprivation.

Statistics

We extracted all potentially relevant variables from the Healthcare Episode Statistics Database (Table 1). Linkage was performed using UK National Health Service numbers which are unique patient identifiers. Given the limited prior information available on risk factors for acquiring a clustered isolate, we considered this analysis to be exploratory. Backwards model selection using the Akaike Information Criteria (AIC) was therefore used to select the candidate final model. Following this, variables were re-entered into the model one at a time. We also re-entered outpatient appointments, inpatient admission days and respiratory procedures as binary variables to check whether this improved the fit of the model (i.e. 1 if >0 outpatient/inpatient attendances/procedures). We tested for potential interactions between all final exposures/confounders in the final model. To allow for multiple testing, we prespecified that interactions would only be considered significant at the p<0.01 level. We used the MFP package in R to determine whether non-linear transformations of continuous variables might improve the fit of the final model. All statistics was performed using R version 3.4.3 and the MASS[14] (for backwards model selection) and Comorbidity[15] packages (for calculating Elixhauser scores). Outcomes were expressed as odds rations/adjusted odds ratios with 95% confidence intervals.

Patients in the same postcode

We acquired postcodes for CF patients who had had at least one positive M. abscessus isolated through linkage with the Health Episode Statistics database and took the postcode closest in time to the date of collection as described above. We then searched the Health Episode Statistics Database for other patients living at the same postcode in the same financial year who had a diagnosis of Cystic Fibrosis using Microsoft SQL Server Management Studio to interface with the Public Health England Data Lake.

Supplementary Results

Additional potential sibling pair

In addition to the three confirmed sibling pairs (identified as living at the same address and sharing a surname), there was a further pair of individual who both had CF, were of a similar age and shared the same postcode for 6 years (these six years started at the beginning of data availability in the Health Episode Statistics database and so the real period is likely to be longer). Both of these individuals acquired M. abscessus around the same time and these strains were highly divergent (54949 SNPs). We classify them only as a possible sibling pair because they did not share the same postcode at the time when they acquired M. abscessus and this was 13 years after they had last shared a postcode. Additionally they had different surnames (though these may have changed by marriage).

References

1. Walker TM, Kohl TA, Omar SV, et al. Whole-genome sequencing for prediction of Mycobacterium tuberculosis drug susceptibility and resistance: a retrospective cohort study. Lancet Infect Dis 2015;15(10):1193–202.

2. Lunter G, Goodson M. Stampy: a statistical algorithm for sensitive and fast mapping of Illumina sequence reads. Genome Res [Internet] 2011;Available from: https://genome.cshlp.org/content/21/6/936.short

3. Li H, Handsaker B, Wysoker A, et al. The Sequence Alignment/Map format and SAMtools. Bioinformatics 2009;25(16):2078–9.

4. Nguyen L-T, Schmidt HA, von Haeseler A, Minh BQ. IQ-TREE: a fast and effective stochastic algorithm for estimating maximum-likelihood phylogenies. Mol Biol Evol 2015;32(1):268–74.

5. Page AJ, Taylor B, Delaney AJ, et al. SNP-sites: rapid efficient extraction of SNPs from multi-FASTA alignments. Microb Genom 2016;2(4):e000056.

6. Didelot X, Wilson DJ. ClonalFrameML: efficient inference of recombination in whole bacterial genomes. PLoS Comput Biol 2015;11(2):e1004041.

7. Didelot X, Croucher NJ, Bentley SD, Harris SR, Wilson DJ. Bayesian inference of ancestral dates on bacterial phylogenetic trees. Nucleic Acids Res 2018;46(22):e134.

8. Drummond AJ, Rambaut A. BEAST: Bayesian evolutionary analysis by sampling trees. BMC Evol Biol 2007;7:214.

9. Harris S. TreeGubbins [Internet]. Github; [cited 2020 Sep 15]. Available from: https://github.com/simonrharris/tree_gubbins

10. Bryant JM, Grogono DM, Rodriguez-Rincon D, et al. Emergence and spread of a human-transmissible multidrug-resistant nontuberculous mycobacterium. Science 2016;354(6313):751–7.

11. Everall I, Nogueira CL, Bryant JM, et al. Genomic epidemiology of a national outbreak of post-surgical Mycobacterium abscessus wound infections in Brazil. Microb Genom 2017;3(5):e000111.

12. Tortoli E, Kohl TA, Trovato A, et al. Mycobacterium abscessus in patients with cystic fibrosis: low impact of inter-human transmission in Italy. Eur Respir J [Internet] 2017;50(1). Available from: http://dx.doi.org/10.1183/13993003.02525-2016

13. Postcodes.io - free postcode lookup API and geocoder for the UK [Internet]. [cited 2020 Aug 19];Available from: https://postcodes.io/

14. Venables WN, Ripley BD. Modern Applied Statistics with S [Internet]. 2002;Available from: http://www.stats.ox.ac.uk/pub/MASS4

15. Gasparini A. comorbidity: An R package for computing comorbidity scores. Journal of Open Source Software 2018;3(23):648.

Supplementary Figures

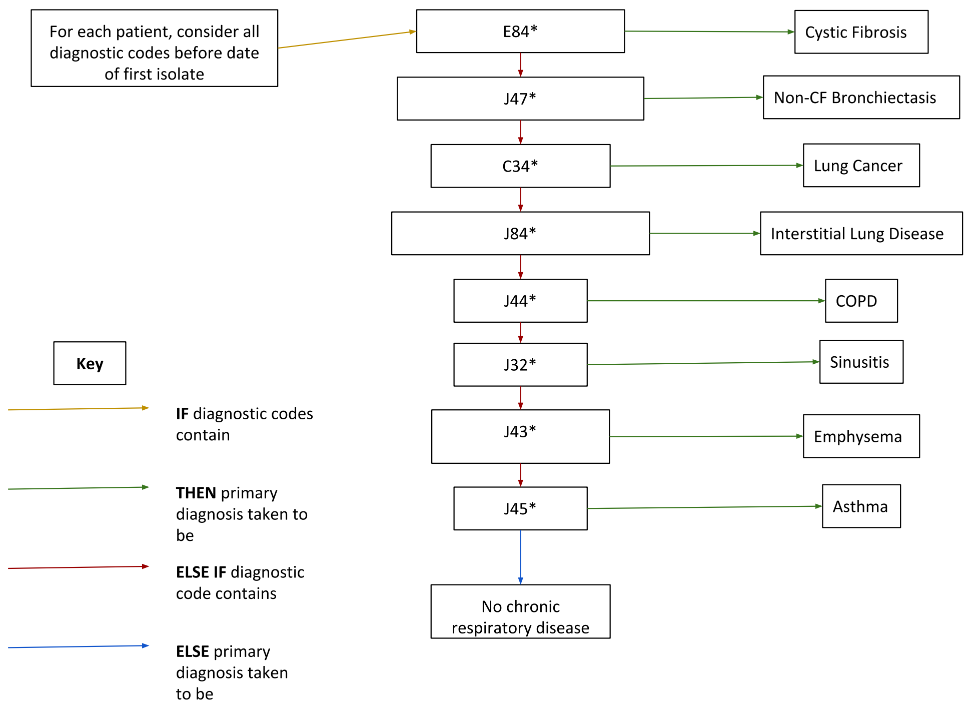

Figure S1: logical algorithm used to assign respiratory diagnoses to patients based on ICD10 codes in the Health Episode Statistics database. * wildcard term.

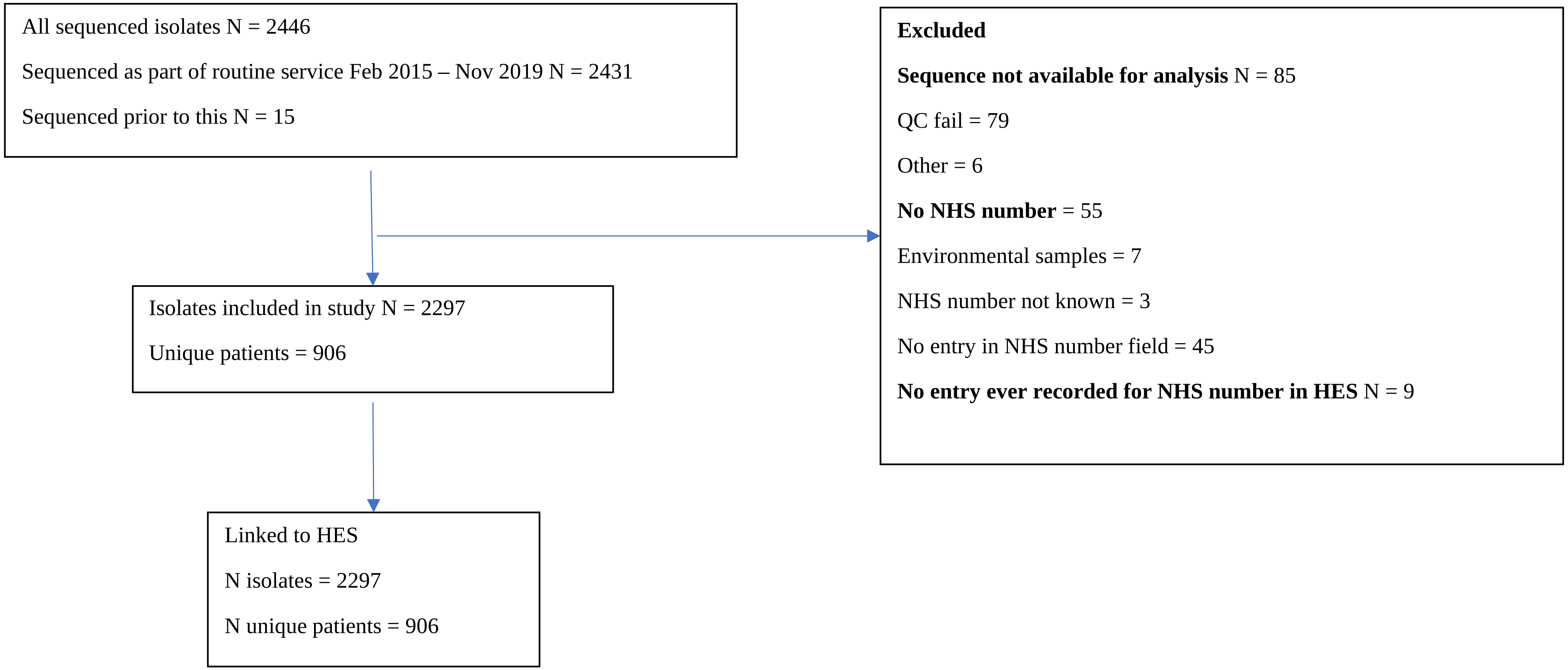

Figure S2: description of isolates included/excluded in the study

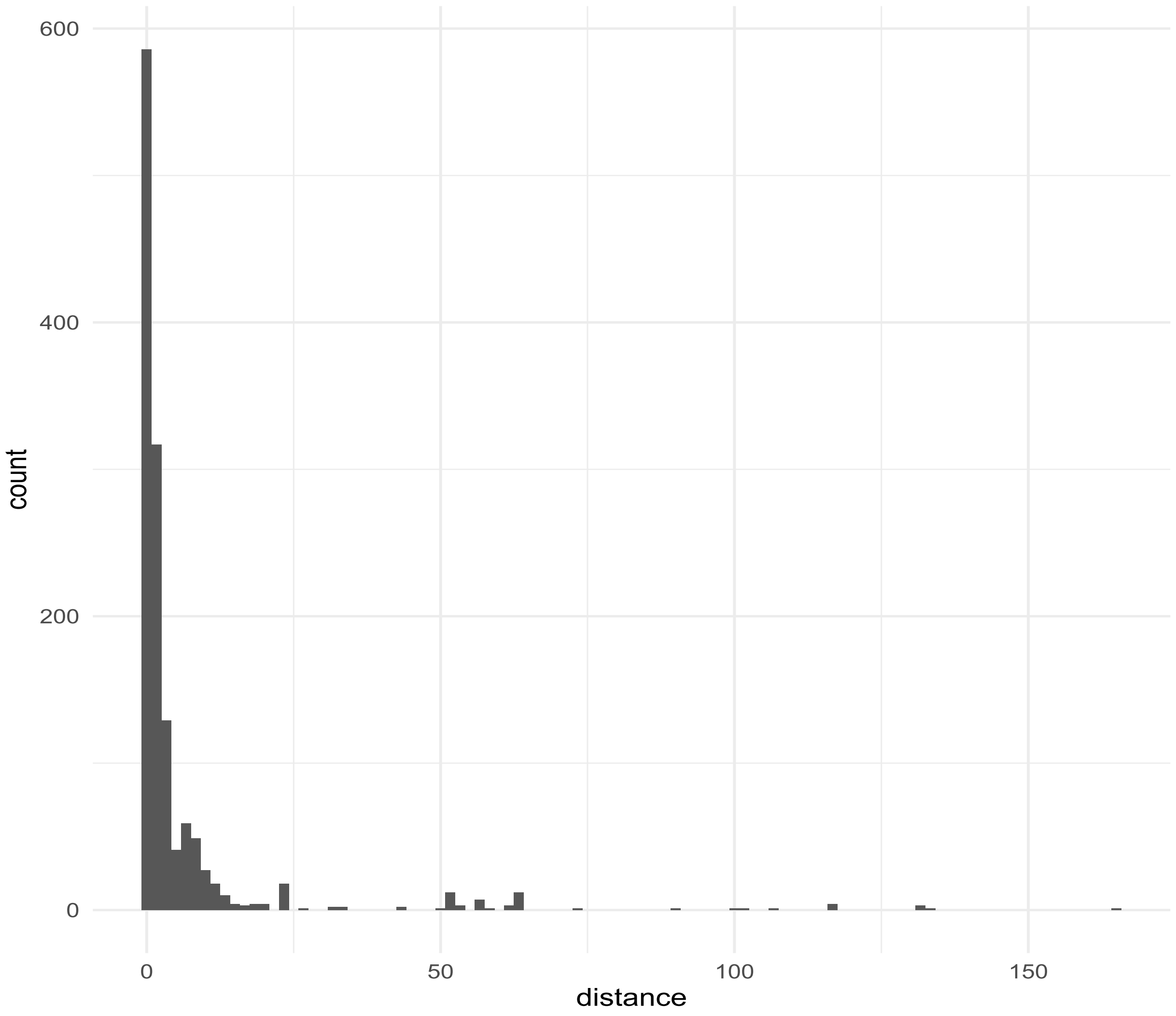

Figure S3: Distribution of within patient genomic distances (single nucleotide polymorphisms) for the same subspecies

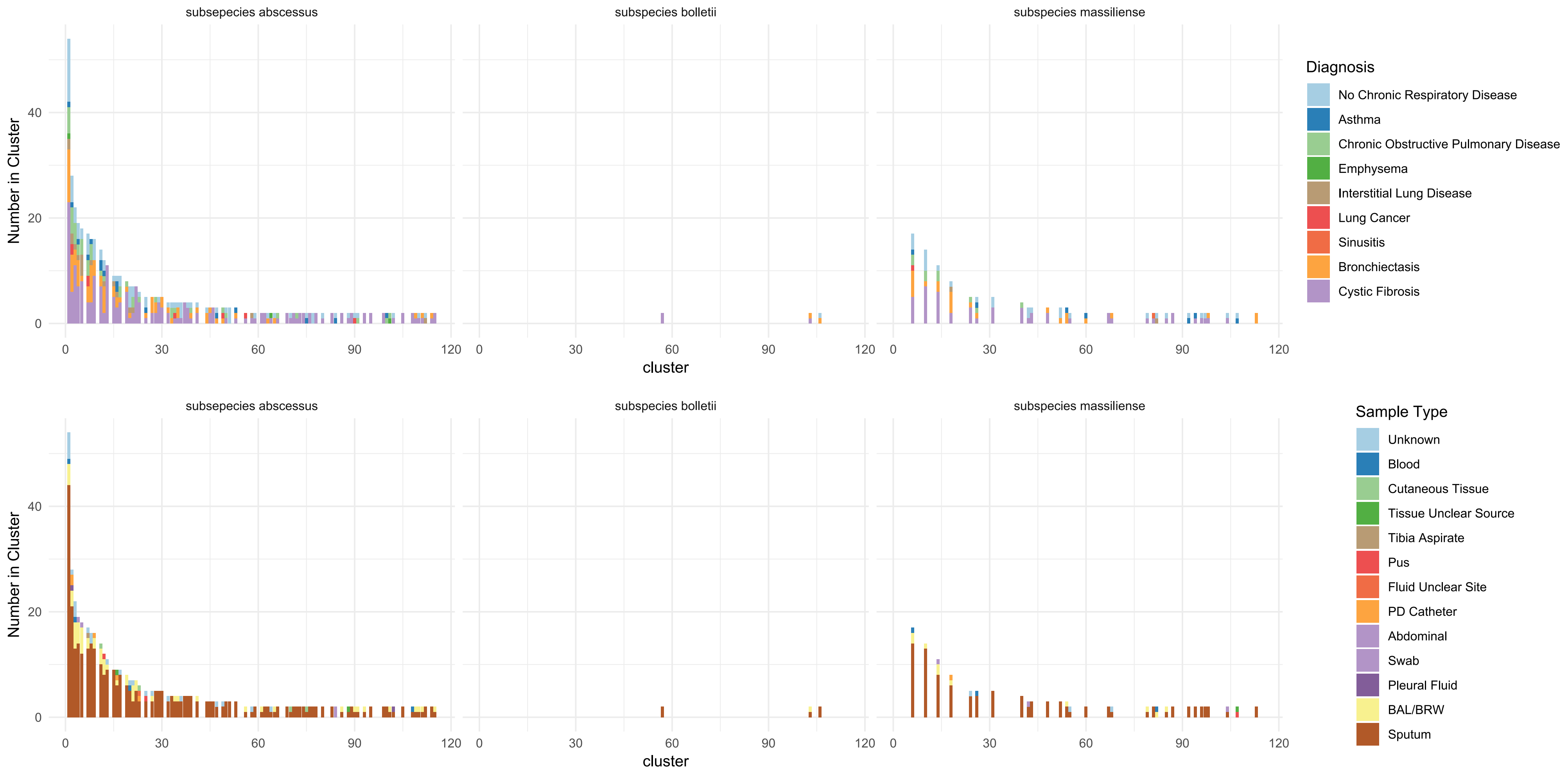

Figure S4: Breakdown of Figure 2 (showing distribution of cluster sizes, coloured by diagnosis (top) and sample type (bottom) by subspecies.

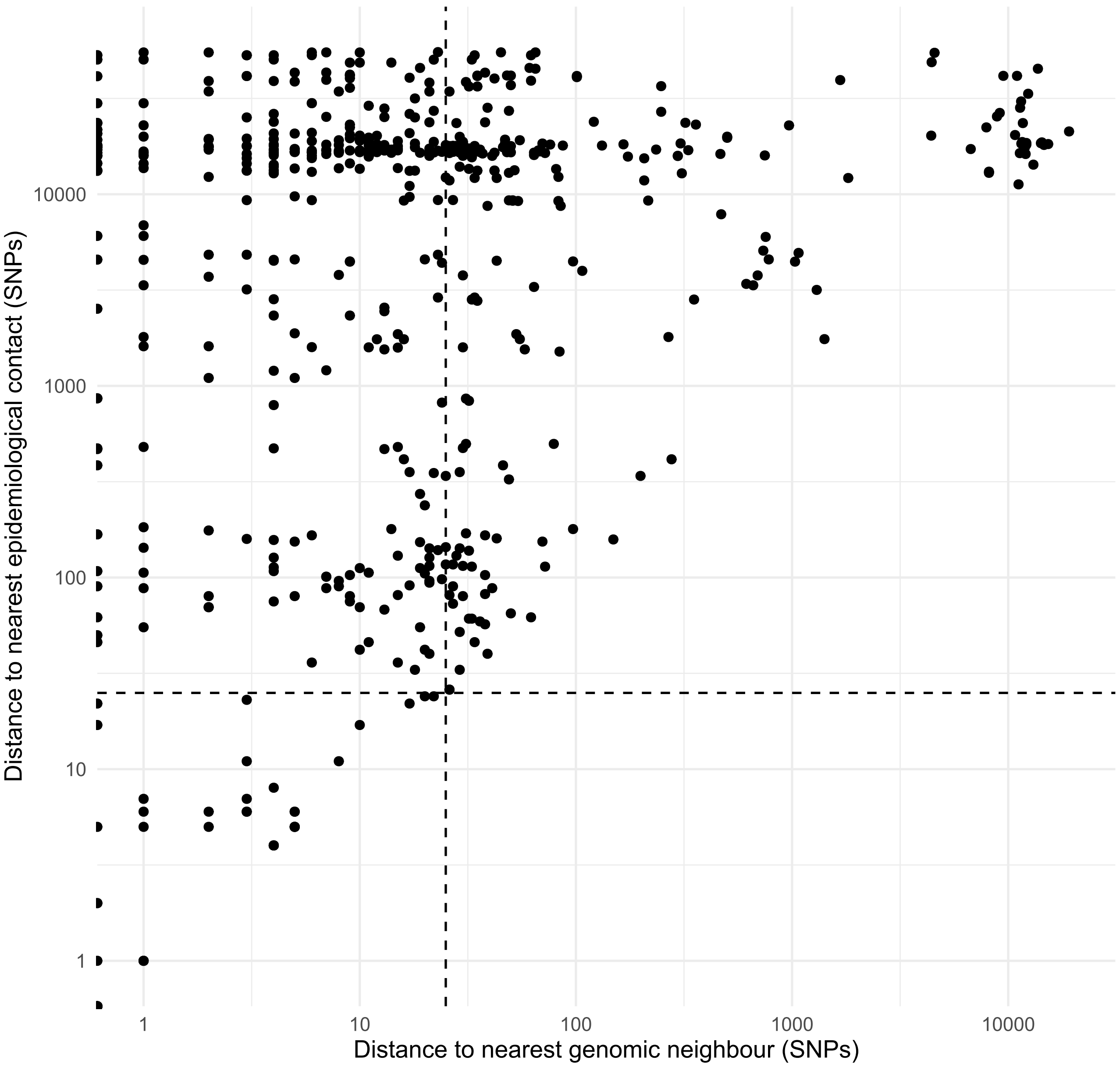

Figure S5: Distance to closest genetic neighbour (Single Nucleotide Polymorphisms (SNPs) plotted against distance to nearest genetic neighbour with which the patient had an epidemiological contact. Hashed lines show the position of x/y = 25 SNPs (i.e. potentially compatible with recent transmission.

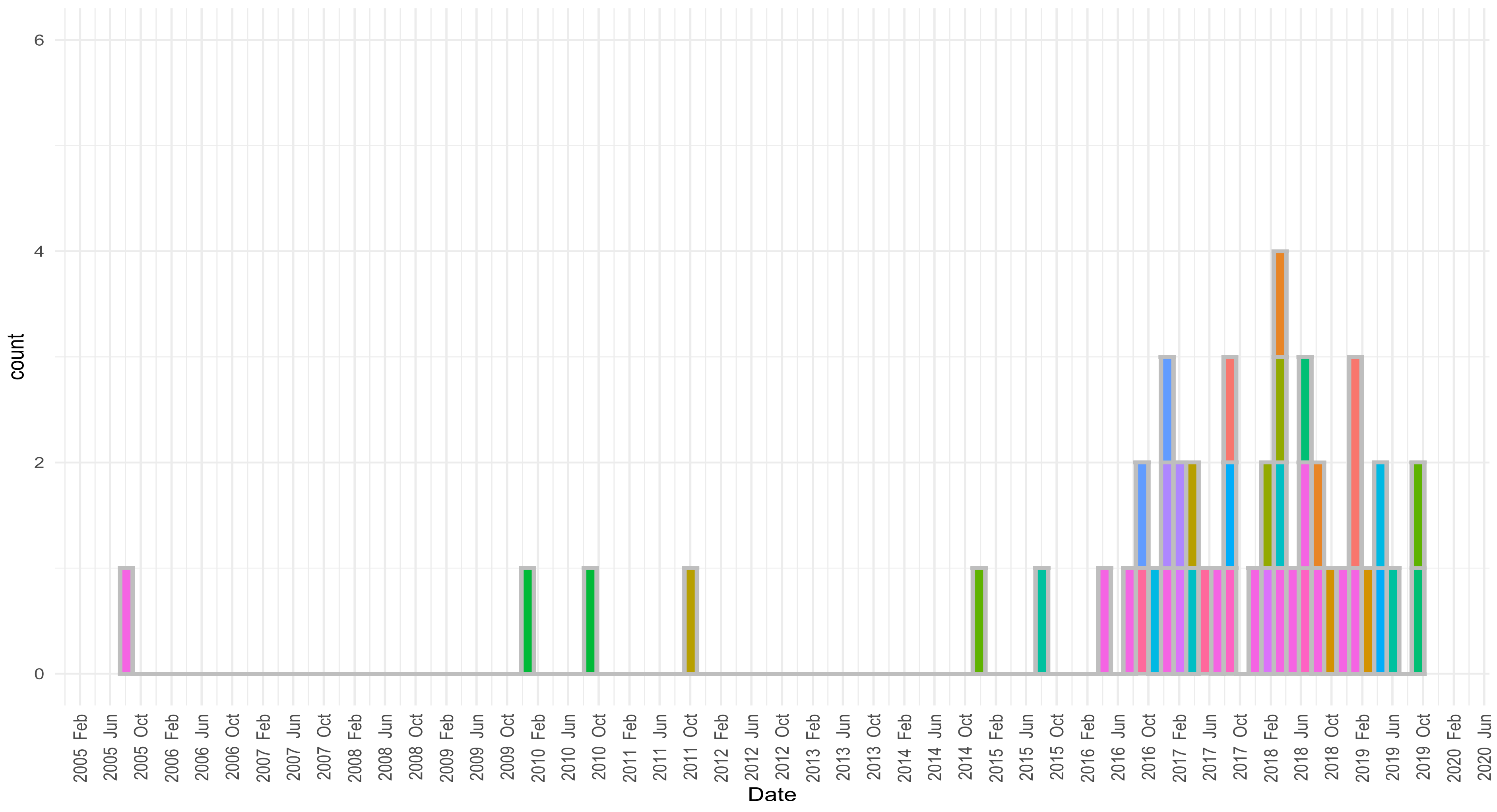

Figure S6: Epidemic curve for isolates with a nearest genomic and nearest epidemiological neighbour within 25 SNPs. Colours represent different clusters.

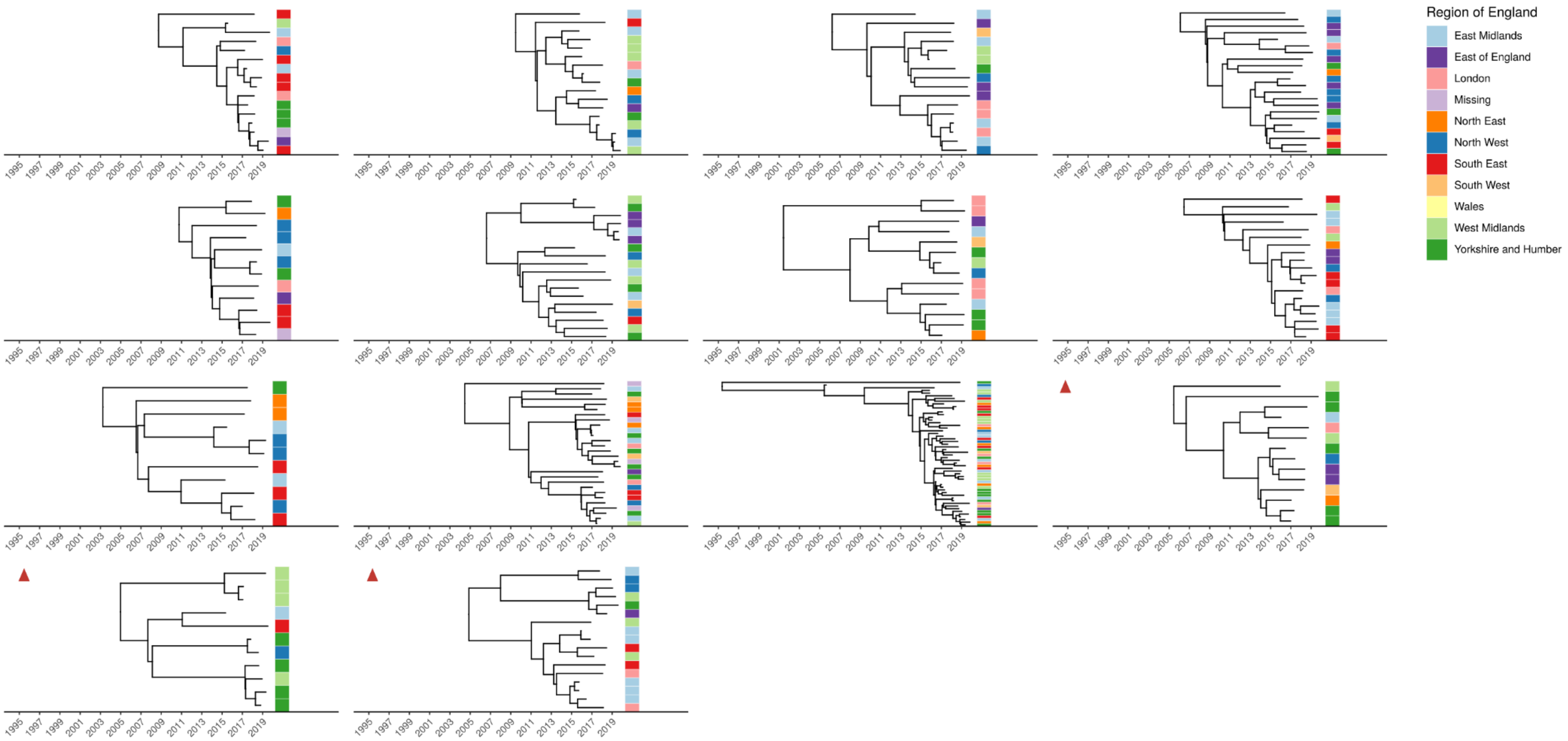

Figure S7: Dated phylogenies for all larger clusters (N > =10, identified using the <25 SNP threshold). The side panel shows the region of England in which the patient lived. Red triangles denote clusters in *M. abscessus* subspecies *massiliense*. All other clusters are *M. abscessus* subspecies *abscessus*.

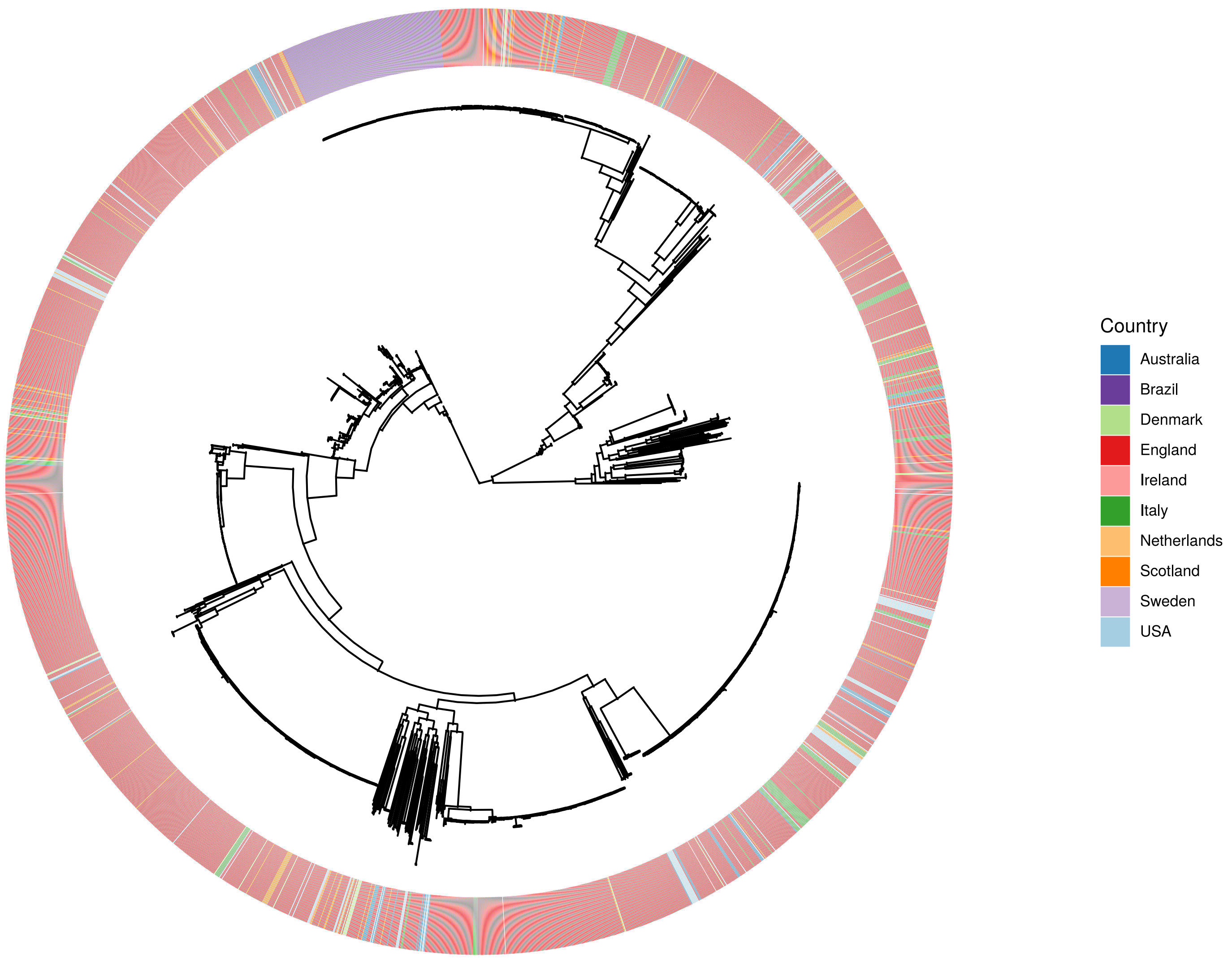

Figure S8: Phylogenetic tree showing distribution of isolates from previous global studies, compared to those from the present study (shown here as ‘England’).

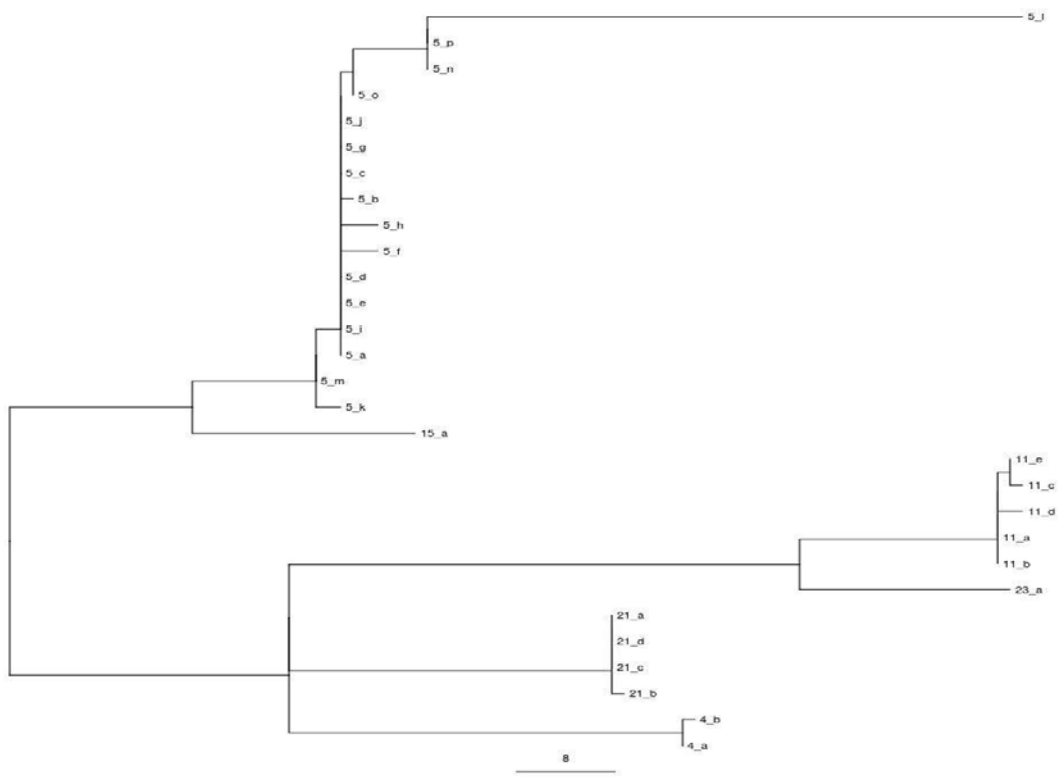

Figure S9: Phylogenetic tree of *M. abscessus subsp massiliense* clusters from a previous UK study mapped to NC 010397.10. Tip labels are those used in the original study. Data was acquired from NCBI accession ERP001039 and the tree was constructed in using the methodology detailed in the supplementary methods. The scale bar represents SNPs per genome.

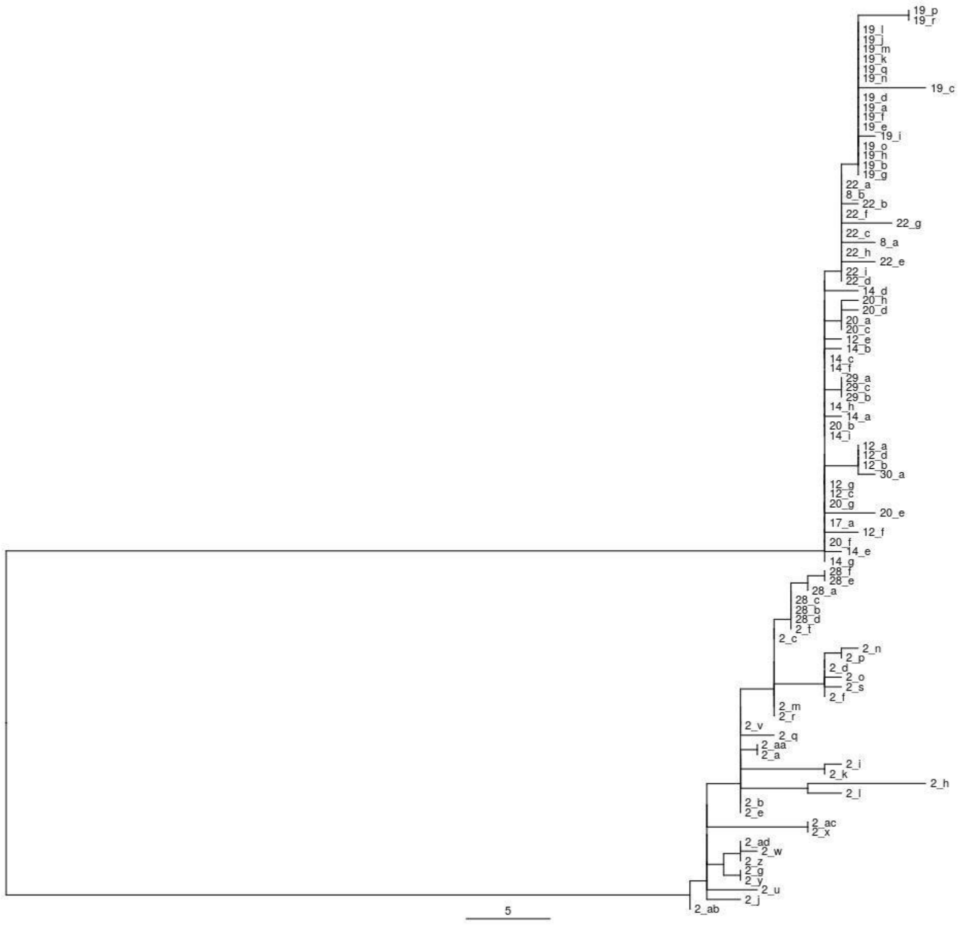

Figure S10: Phylogenetic tree of *M. abscessus subsp massiliense* clusters from a previous UK study mapped to NC 010397.10. Tip labels are those used in the original study. Data was acquired from NCBI accession ERP001039 and the tree was constructed in using the methodology detailed in the supplementary methods. The scale bar represents SNPs per genome. Despite our choice to map to a single *M. abscessus abscessus subsp. abscessus* reference, clusters are identical to those identified in the original study.

Supplementary Tables

Sample type	N
Abdominal	4
Aspirate Unclear Site	2
Bronchoalveolar Lavage/Bronchial Washing	135
Blood	19
Breast	4
Chin Implant	1
Cutaneous Tissue	10
Fluid Unclear Site	4
Lung Tissue	2
Lymph Biopsy	1
Peritoneal Dialysis Catheter	8
Pleural Fluid	3
Pus	9
Spinal Aspirate	1
Sputum	1997
Swab	8
Synovial Fluid	3
Tibia Aspirate	1
Tissue Unclear Source	14
Unknown	71

Table S1: Number of each sample type for the 2297 isolates in the study.

Variable		Bronchiectasis	No Chronic Respiratory Disease	Asthma	Lung Cancer	Cystic Fibrosis	Chronic Obstructive Pulmonary Disease	Interstitial Lung Disease
Gender – N (%)	F	93 (62.0)	113 (55.9)	15 (39.5)	4 (40.0)	180 (44.1)	25 (33.8)	12 (50.0)
	M	57 (38.0)	87 (43.1)	21 (55.3)	6 (60.0)	227 (55.6)	48 (64.9)	11 (45.8)
	U		2 (1.0)	2 (5.3)		1 (0.2)	1 (1.4)	1 (4.2)
Age - Median (IQR)		70 (59-77)	55 (34.8-70.2)	61.5 (46.2-70)	73.5 (68.2-78.2)	21 (16-27)	71 (65-78.2)	64 (58-76.5)
Outpatient Attendances - Median (IQR)		8 (4-14)	5 (1-12)	7 (2.3-15.8)	15.5 (5.3-21)	12 (8-16)	7 (3-12.8)	10 (5-19.2)
Inpatient Days - Median (IQR)		0 (0-5)	0 (0-3)	0 (0-1)	10 (2.8–22.8)	7 (0-20)	2 (0-10.8)	2.5 (0-16)
Elixhauser Score - Median (IQR)		7 (3-13)	0 (0-5)	3 (3-8.8)	19 (13-22.5)	3 (0-11)	9 (3-17)	6.5 (3.8-13.2)
Respiratory Procedures - Median (IQR)		0 (0-0)	0 (0-0)	0 (0-0)	1 (1-2.75)	0 (0-0)	0 (0-1)	1 (0-1)
Rural/Urban dwelling – N (%)	Hamlet	7 (4.7)	3 (1.5)	1 (2.6)	0 (0)	10 (2.5)	0 (0)	1 (4.3)
	Town and Fringe	13 (8.7)	8 (4.0)	2 (5.3)	0 (0)	45 (11.1)	11 (15.1)	1 (4.3)
	Urban	118 (79.2)	174 (86.6)	32 (84.2)	10 (100.0)	319 (78.4)	59 (80.8)	20 (87.0)
	Village	11 (7.4)	15 (7.5)	3 (7.9)		32 (7.9)	3 (4.1)	1 (4.3)
	Unknown	0 (0)	1 (0.5)	0 (0)	0 (0)	1 (0.2)	0 (0)	0 (0)
Index of Multiple Deprivation Decile N (%)	Most deprived 10%	14 (9.5)	26 (14.1)	5 (13.9)	1 (10.0)	39 (9.7)	14 (18.9)	4 (16.7)
	More deprived 10-20%	12 (8.1)	25 (13.5)	7 (19.4)	2 (20.0)	44 (10.9)	12 (16.2)	2 (8.3)
	More deprived 20-30%	11 (7.4)	16 (8.6)	4 (11.1)	2 (20.0)	40 (9.9)	7 (9.5)	4 (16.7)
	More deprived 30-40%	14 (9.5)	12 (6.5)	4 (11.1)	1 (10.0)	33 (8.2)	3 (4.1)	0 (0.0)

Variable		Bronchiectasis	No Chronic Respiratory Disease	Asthma	Lung Cancer	Cystic Fibrosis	Chronic Obstructive Pulmonary Disease	Interstitial Lung Disease
	More deprived 40-50%	17 (11.5)	21 (11.4)	0 (0.0)	1 (10.0)	45 (11.2)	9 (12.2)	3 (12.5)
	Less deprived 50-60%	11 (7.4)	18 (9.7)	2 (5.6)	0 (0.0)	32 (7.9)	7 (9.5)	0 (0.0)
	Less deprived 60-70%	14 (9.5)	12 (6.5)	3 (8.3)	0 (0.0)	33 (8.2)	8 (10.8)	3 (12.5)
	Less deprived 70-80%	16 (10.8)	18 (9.7)	6 (16.7)	2 (20.0)	41 (10.2)	4 (5.4)	1 (4.2)
	Less deprived 80-90%	19 (12.8)	16 (8.6)	4 (11.1)	1 (10.0)	60 (14.9)	4 (5.4)	2 (8.3)
	Least deprived 10%	20 (13.5)	21 (11.4)	1 (2.8)	0 (0.0)	36 (8.9)	6 (8.1)	5 (20.8)

Table S2 – Characteristics of the 906 patients included in the study. Missing data: Sex n=7 (shown as Unknown), Age n=6, Index of Multiple Deprivation Decile n=26, Rural/Urban Indicator n=5. * Inpatient Days/Outpatient Attendances/Respiratory Procedures refer to the number of these in the year before M. abscessus was first isolated from the patient.

Sample Type	Not clustered	Clustered
Abdominal	0	4
Aspirate Unclear Site	1	0
BAL/BRW	19	67
Blood	2	7
Breast	3	0
Chin Implant	1	0
Cutaneous Tissue	2	4
Fluid Unclear Site	2	1
Lung Tissue	2	0
Lymph Biopsy	1	0
PD Catheter	1	6
Pleural Fluid	0	2
Pus	2	3
Sputum	309	452
Swab	1	3
Tibia Aspirate	0	1
Tissue Unclear Source	7	3
Unknown	11	27

Table S3: Sample types of the 944 isolates (retaining one genome per patient per cluster). Isolates were clustered using the < 25 SNP threshold.

Variable		Not Clustered N (%)/Median (IQR)	Clustered N (%)/Median (IQR)	OR (univariable)	OR (multivariable)
Gender	F	71 (39.7)	108 (60.3)	-	-
	M	94 (42.5)	127 (57.5)	0.89 (0.59-1.33, p=0.56)	
Age	Mean (SD)	20 (17-25)	21 (15-27)	1.01 (0.99-1.04, p=0.24)	
Outpatient Attendances	Mean (SD)	12 (7-18)	12 (8-16)	1.00 (0.98-1.02, p=0.73)	
Inpatient days (per 7 days)	Mean (SD)	7 (0-22)	7 (0-17.5)	0.99 (0.99-1.00, p=0.19)	0.94 (0.88-1.00, p=0.04)
Elixhauser Score	Mean (SD)	3 (0-11)	5 (0-11.5)	1.02 (0.99-1.05, p=0.12)	1.03 (1.00-1.06, p=0.06)
Respiratory Procedures	Mean (SD)	0 (0-0)	0 (0-1)	1.13 (0.93-1.42, p=0.24)	1.18 (0.96-1.49, p=0.14)
Rural/Urban Dwelling	Hamlet	4 (40.0)	6 (60.0)	-	-
	Town and Fringe	23 (52.3)	21 (47.7)	0.61 (0.14-2.43, p=0.49)	
	Urban	122 (38.7)	193 (61.3)	1.05 (0.27-3.77, p=0.94)	
	Village	16 (51.6)	15 (48.4)	0.63 (0.14-2.62, p=0.53)	
Index of Multiple Deprivation Decile	More deprived 10-20%	19 (43.2)	25 (56.8)	-	-
	More deprived 20-30%	15 (37.5)	25 (62.5)	1.27 (0.53-3.07, p=0.60)	
	Less deprived 50-60%	12 (37.5)	20 (62.5)	1.27 (0.50-3.26, p=0.62)	
	Most deprived 10%	16 (41.0)	23 (59.0)	1.09 (0.46-2.63, p=0.84)	
	More deprived 40-50%	22 (48.9)	23 (51.1)	0.79 (0.34-1.83, p=0.59)	
	Least deprived 10%	15 (42.9)	20 (57.1)	1.01 (0.41-2.50, p=0.98)	
	Less deprived 70-80%	18 (43.9)	23 (56.1)	0.97 (0.41-2.30, p=0.95)	
	Less deprived 80-90%	28 (48.3)	30 (51.7)	0.81 (0.37-1.79, p=0.61)	
	Less deprived 60-70%	8 (24.2)	25 (75.8)	2.37 (0.90-6.70, p=0.09)	
	More deprived 30-40%	12 (36.4)	21 (63.6)	1.33 (0.53-3.41, p=0.55)	

Table S4: Multivariable predictors of having a clustered isolate in patients with Cystic Fibrosis (CF). Univariable estimates are shown for all variables, multivariable estimates are only shown for variables included in the final model.

High-density cluster number	N patients	N NUTS regions	Median SNP distance between isolates (IQR)	Medium SNP distance between isolates in same NUTS region (IQR)	Medium SNP distance between isolates in different NUTS region (IQR)	Observed SNP ratio	Expected random SNP ratio (permuted)
1	90	9	120 (76 - 165)	121 (80 - 163)	120 (76 - 166)	1	0.9 - 1.1
2	138	9	149 (93 - 182)	135 (85 - 183)	141 (94 - 182)	1	0.9 - 1.1
3	82	9	32 (7 - 472)	40 (7 - 474)	31 (7 - 472)	1.3	0.5 - 3.4
4	41	9	47 (31 - 67)	45 (28 - 65)	47 (31 - 67)	1	0.8 - 1.2
5	124	9	2315 (1299 - 3171)	2243 (1219 - 3168)	2321 (1303 - 3172)	1	0.9 - 1.0
6	33	9	65 (32 - 127)	72 (36 - 128)	65 (31 - 127)	1.1	0.6 - 1.8
7	40	8	303 (98 - 371)	275 (94 - 363)	313 (98 - 372)	0.9	0.5 - 1.2
8	18	7	28 (14 - 25)	18 (12 - 32)	18 (14 - 25)	1	0.8 - 1.3

Table S5: Within NUTS region clustering by high-density phylogenetic cluster. IQR – interquartile range
